# Supplementary material for: SMS messaging to improve retention and viral suppression in prevention of mother-to-child HIV transmission (PMTCT) programs in Kenya: A 3-arm randomized clinical trial
Source: PLoS Med. 2021 May 24;18(5):e1003650. doi: 10.1371/journal.pmed.1003650 (PMC8186790; doi:10.1371/journal.pmed.1003650)
Supplement: S1 Table — (DOCX) [file pmed.1003650.s002.docx]

**Supplementary Table 1. Incidence rate of virologic non-suppression by arm**

| **Anderson-Gill regression** | **Overall (N=824)** | **Control (N=277)** | **One-way (N=271)** | **Two-way (N=276)** | **One-way vs control** | **Two-way vs control** | **Either arm vs control** | **Two-way vs one-way** |
| --- | --- | --- | --- | --- | --- | --- | --- | --- |
| IR of VF by delivery (per 100 p-years)  IR of VF by 180 days pp (per 100 p-years)  IR of VF by 365 days pp (per 100 p-years)  IR of VF by 730 days pp (per 100 p-years)  IR of VF by 850 days pp (per 100 p-years)  IR of VF by all days pp (per 100 p-years) | 15/13 (115.4)  74/148 (50.0)  147/393 (37.4)  224/879 (25.5)  265/1121 (23.6)  304/1448 (20.1) | 3/4 (75.0)  24/47 (51.1)  45/124 (36.2)  73/289 (25.2)  85/368 (23.1)  97/492 (19.7) | 8/5 (160.0)  35/50 (70.0)  65/136 (47.8)  89/292 (30.5)  102/363 (28.1)  118/469 (25.2) | 4/4 (100.0)  15/52 (28.8)  37/133 (27.8)  62/298 (20.8)  78/390 (20.0)  89/487 (18.3) | cHR: 1.44 (0.19-10.93), p=0.73  aHR: 1.46 (0.20-10.73), p=0.71  cHR: 1.37 (0.74-2.54), p=0.32  aHR: 1.33 (0.73-2.43), p=0.36  cHR: 1.28 (0.79-2.08), p=0.31  aHR: 1.25 (0.77-2.04), p=0.37  cHR: 1.21 (0.79-1.86), p=0.38  aHR: 1.18 (0.77-1.82), p=0.46  cHR: 1.24 (0.82-1.86), p=0.31  aHR: 1.18 (0.78-1.78), p=0.44  cHR: 1.28 (0.87-1.90), p=0.24  aHR: 1.22 (0.82-1.81), p=0.32 | cHR: 0.96 (0.12-7.47), p=0.97  aHR: 0.97 (0.12-7.68), p=0.97  cHR: 0.56 (0.26-1.21), p=0.14  aHR: 0.55 (0.26-1.17), p=0.12  cHR: 0.75 (0.43-1.28), p=0.29  aHR: 0.73 (0.43-1.25), p=0.26  cHR: 0.83 (0.51-1.36), p=0.45  aHR: 0.80 (0.50-1.30), p=0.38  cHR: 0.86 (0.54-1.36), p=0.52  aHR: 0.82 (0.52-1.28), p=0.39  cHR: 0.93 (0.61-1.44), p=0.76  aHR: 0.89 (0.58-1.36), p=0.60 | cHR: 1.22 (0.18-8.40), p=0.84  aHR: 1.24 (0.18-8.39), p=0.82  cHR: 0.96 (0.53-1.72), p=0.88  aHR: 0.93 (0.52-1.65), p=0.81  cHR: 1.00 (0.64-1.55), p=0.98  aHR: 0.99 (0.64-1.53), p=0.96  cHR: 1.02 (0.69-1.50), p=0.93  aHR: 0.99 (0.67-1.46), p=0.96  cHR: 1.04 (0.72-1.50), p=0.83  aHR: 0.99 (0.69-1.43), p=0.96  cHR: 1.11 (0.78-1.57), p=0.58  aHR: 1.06 (0.74-1.50), p=0.76 | cHR: 0.67 (0.19-2.40), p=0.54  aHR: 0.66 (0.19-2.31), p=0.52  cHR: 0.41 (0.20-0.82), **p=0.01**  aHR: 0.41 (0.21-0.82), **p=0.01**  **cHR: 0.58 (0.35-0.97), p=0.04**  **aHR: 0.59 (0.35-0.97), p=0.04**  cHR: 0.68 (0.42-1.11), p=0.12  aHR: 0.68 (0.42-1.10), p=0.12  cHR: 0.69 (0.44-1.09), p=0.12  aHR: 0.70 (0.45-1.09), p=0.12  cHR: 0.73 (0.48-1.12), p=0.15  aHR: 0.73 (0.48-1.11), p=0.14 |

cHR: crude hazard ratio, compared by Andersen-Gill regression

aHR: adjusted for primigravida & employment, compared by Andersen-Gill regression

Key for numbers in the first 4 columns N=number of participants; each cell contains # events / person-years (events/100 pyears)
