# Supplementary figures and images for: SMS messaging to improve retention and viral suppression in prevention of mother-to-child HIV transmission (PMTCT) programs in Kenya: A 3-arm randomized clinical trial
Source: PLoS Med. 2021 May 24;18(5):e1003650. doi: 10.1371/journal.pmed.1003650 (PMC8186790; doi:10.1371/journal.pmed.1003650)

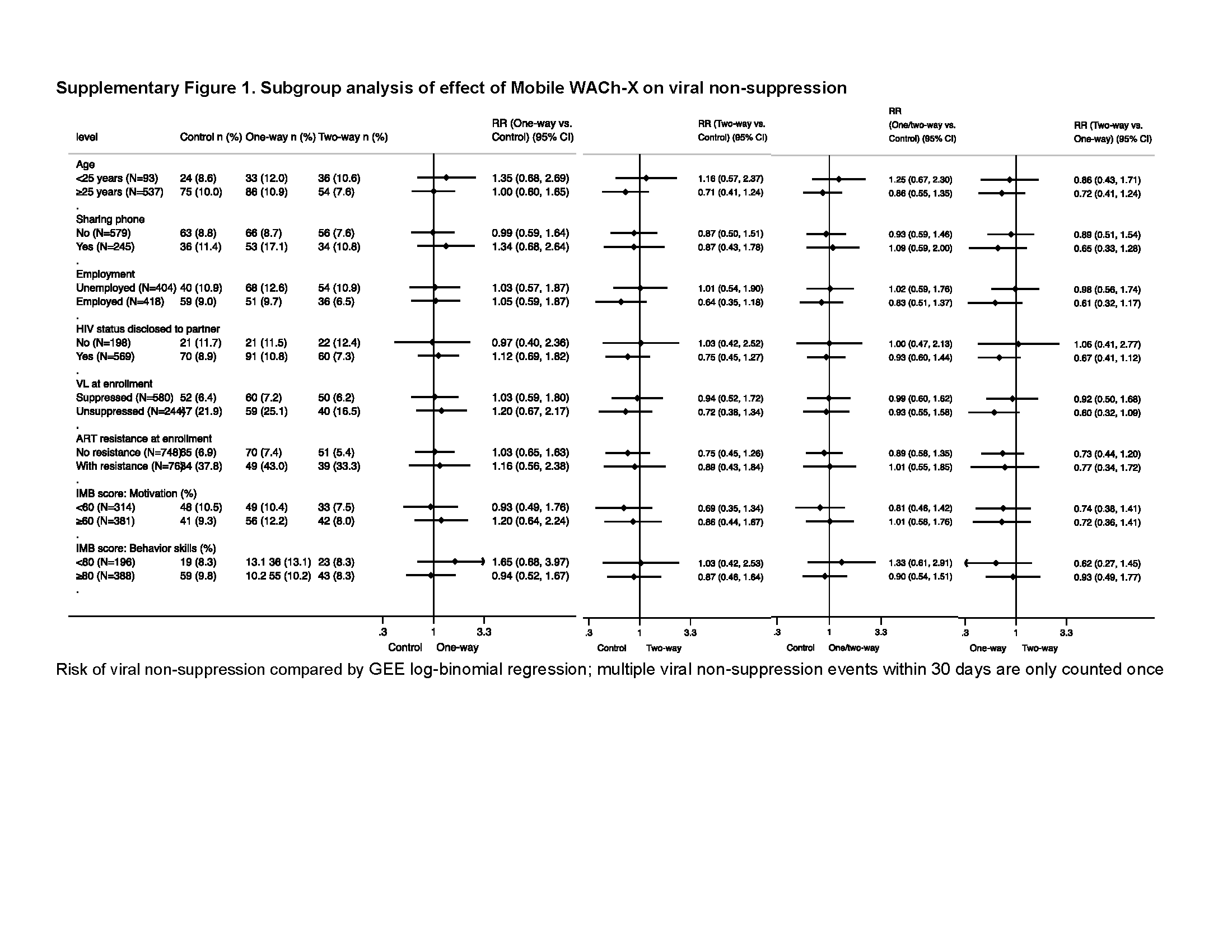

Supplement: S1 Fig — ART, antiretroviral therapy; CI, confidence interval; GEE, generalized estimating equations; IMB, information–motivation–behavior skills; RR, relative risk; VL, viral load. (TIF) [file pmed.1003650.s003.tif]
