# Supplementary material for: SMS messaging to improve retention and viral suppression in prevention of mother-to-child HIV transmission (PMTCT) programs in Kenya: A 3-arm randomized clinical trial
Source: PLoS Med. 2021 May 24;18(5):e1003650. doi: 10.1371/journal.pmed.1003650 (PMC8186790; doi:10.1371/journal.pmed.1003650)
Supplement: Attachment — Submitted filename: PloS Med review - SMS for post-partum ART viral suppression April 2021.docx [file pmed.1003650.s004.docx]

Overall, this was a good paper that was generally clearly written, and with a thoughtful discussion. However, the study size was not large, and greater critique of the assumptions made for the study power calculation would be helpful.

Some specific comments, by section, are given below.

**Abstract**

1. Time on ART 1 year – does this mean follow-up from starting to 1 year after? Or on ART for 1 year at time of recruitment? It becomes clear in the main text, but needs to be clearer in the abstract.

2. Should report in the abstract the absolute values of the outcomes (percentages), and always by trial arm. For the purpose of the study, it is not so helpful to present figures pooled across trial arms and then say there was no difference by trial arm but not showing this.

3. Related to the issue of study power, for which more detail should be provided in the methods of the main paper: the RR of 0.8 comparing 2-way SMS vs control for “ever virally non-suppressed” has a wide confidence interval, and it would be better to say “no evidence of a difference” rather than “no difference” compared to the control arm. I think this result does not correspond to the study power calculation, which was for the cumulative incidence of viral non-suppression by 24 months? It is important to present, as the first result, the one that corresponded to the primary study question for which the study was powered.

4. Need to quote figures on the time to postpartum contraception, especially as the conclusion says the SMS made a difference –but not quantified in the results.

5. Not sure how the conclusion about targeted SMS follows from the results that are presented, the logic for this conclusion could be clarified in relation to the results presented.

**Introduction**

A bit more detail on the evidence to date on SMS to support adherence to ART post-partum would be helpful, beyond that there is “promising, but mixed evidence” and two papers cited. However, both the papers are systematic reviews – the introduction could mention that there are 2 systematic reviews, and then give a bit more information on what they found and whether there were patterns to the heterogeneity found e.g. did the SMS work better in particular settings, for particular groups of women, when implemented in particular ways?

**Methods**

1. Study power is very important here, given the study size is not large for a 3-arm trial and given the absence of evidence for effect on the primary outcomes. It seems from the methods text that the study was powered to be able to show a difference between 75% viral suppression in the control arm and 85% or more in the intervention arm, for a comparison of 2-year postpartum virologic non-suppression or retention. It could help to express this also as a risk ratio, given the analysis is done with risk (or rate) ratios, e.g. 15/25 = 0.6 so the study was powered to show a risk ratio of 0.6 (and then the risk ratios observed, were more of the order of 0.8 or 1/0.8 or closer to 1).

Please clarify if this outcome was “virologic non-suppression at the 2 year time point” or “any virologic suppression during 2 years of follow-up”. This matters, for which results correspond directly to the study power calculation. Please clarify exactly which analysis in the results corresponds to this power calculation (make sure the language in the results aligns precisely with the language for the study power calculation).

2. Page 14 – says qualitative evaluation of participant experiences will be reported elsewhere, but something would be useful in this paper to explain lack of evidence of effect of the SMS. Please consider including some reference to what was learnt from the qualitative evaluation, in the discussion even if not in the results.

3. Page 15 – Please explain a bit more on “Anderson-Gill regression”. Cumulative incidence could have been estimated using Kaplan-Meier methods and using Cox regression to compare trial arms; please explain how “Anderson-Gill regression” is different for a “time to event” analysis and why it was used instead of Kaplan-Meier estimates of the percentage with the outcome by different time points and Cox regression to estimate rate ratios. There is a footnote/caption to Figure 2 which explains why Anderson-Gill regression was used, and that it gave similar hazard ratios to Cox regression of “time to first event”, but this information should be in the Methods.

**Results**

1. Please present first the results that correspond directly to the study power calculation. It is reported in the first paragraph of the “outcomes of RCT” paragraph that overall 20% were ever non-suppressed by 2 years – if this is the primary outcome, please report it in the text separately by trial arm. But, in Table 2 the focus is on (1) viral non-suppression at any time point (regardless of time point) and (2) viral suppression by particular time points i.e. 6, 12, 24 months post-partum.

Please consider some re-ordering of the presentation of results, to first report on the primary outcomes defined in the study power calculation, and then to present other results that are informative.

2. Please clarify how the numerators and denominators are counted for outcome (2) [viral suppression by particular time points] in Table 2, as the model used is a log-binomial model; is the denominator for the 24-month time point all individuals who were followed up at 24 months?

3. Confidence intervals are in general wide, e.g. aRR 0.80 [0.52-1.23], p=0.31 for comparing 2-way SMS with control for non-suppression over time (page 20). The wide confidence intervals, and how they relate to study power, could be given more attention in the discussion.

4. Page 20 – Outcomes of RCT. Please report in the text the percentage with cumulative incidence of virologic suppression by 24 months, as well as the risk ratios. There are numerators and denominators for the log-binomial model used to estimate cumulative incidence of viral non-suppression in Table 2, but it is not completely clear how these numbers are counted – who contributes to the denominator, and who contributes to the numerator? – it is important that is clear in both Table 2 (e.g. with footnotes for more detail) and in the text.

5. Was it considered to use the Kaplan-Meier method, rather than a log-binomial model, for a simple and transparent unadjusted analysis of the comparison of cumulative incidence of viral non-suppression by trial arm that allows for loss to follow-up? And then to use Cox regression to estimate hazard ratios to compare trial arms? It would help to understand the choice of the log-binomial model, if the numerators and denominators were precisely defined for each time point (who contributes to the analysis), see comment also above.

Figure 2A shows “time to first non-suppression”, I think from a Kaplan-Meier analysis, but then presents hazard ratios from Anderson-Gill regression that allow for repeated instances of viral non-suppression. I think it would be better for Figure 2A to show the hazard ratios from Cox regression, that directly correspond to the graph. The findings from the Anderson-Gill regression could be included in a table instead – they should be included as part of Table 2, and I think they correspond closely to the primary analysis defined for the study power calculation (cumulative incidence of viral suppression by 24 months) but are not exactly the same as they allow for repeated events (of viral non-suppression).

6. In Table 2, please write out what RR stands for in the table column heading, as it matters for interpretation whether is a risk ratio (which I think it is) or a rate ratio and it needs to be clear for the reader and correspond to the analysis that was done. I think from the log-binomial models, it is a risk ratio, but for example if Cox regression were done it would be a rate ratio / hazard ratio.
