# Supplementary material for: SMS messaging to improve retention and viral suppression in prevention of mother-to-child HIV transmission (PMTCT) programs in Kenya: A 3-arm randomized clinical trial
Source: PLoS Med. 2021 May 24;18(5):e1003650. doi: 10.1371/journal.pmed.1003650 (PMC8186790; doi:10.1371/journal.pmed.1003650)
Supplement: Attachment — Submitted filename: ResponseToReviewers_final.docx [file pmed.1003650.s005.docx]

April 7, 2021

Dear Dr. Turner,

Re: PMEDICINE-D-21-00822R1

Thank you for your careful review of our manuscript. We are grateful to the reviewers for their very helpful comments and are gratified that they found our study was “well-written”, “well-conducted”, and “important”.

We have addressed the reviewers’ and editors’ comments below.

Requests from the editors:

In the article metadata, it appears that the information under "competing interests" should be moved to the "funding" section.

We have removed the competing interests.

Please add "in Kenya" to the title, preceding the colon.

This has been added (page 1).

Please quote study dates in your abstract.

This has been added (page 2).

Please combine the "Methods" and "Findings" subsections of your abstract; and add a new final sentence to the restructured combined subsection, which should begin "Study limitations include ..." or similar and quote 2-3 of the study's main limitations.

This has been added (page 2).

Please quote the trial registration number with your abstract, but remove information on funding.

This has been corrected (page 2).

After the abstract, please add a new and accessible "Author summary" section in non-identical prose. You may find it helpful to consult one or two recent research papers in PLOS Medicine to get a sense of the preferred style.

This has been added (page 3).

Please remove trademarks, e.g., in your Methods section.

Trademarks have been removed (page 8, 4^th^ paragraph of procedures section of methods).

Please remove "data not shown" from your Results, and incorporate the relevant data in supplementary files.

We now report all findings (page 20, 3^rd^ from last paragraph of results).

Please remove information on competing interests and study funding (inc. grant numbers) from the end of the main text. In the event of publication, this information will appear in the article metadata, via entries in the submission form.

This has been removed (page 24, acknowledgements section).

Throughout the text, please style reference call-outs as follows: "... in Kenya [11,12]. We hypothesized ..." (noting the absence of spaces within the square brackets).

This has been corrected throughout.

In the reference list, please convert all boldface text and italics into plain text. Where appropriate, 6 author names should be listed rather than 3, followed by "et al.".

This has been corrected.

Does reference 18 lack a journal name?

This has been corrected.

Please rename figure 1 "Participant flowchart" or similar.

This has been corrected and Figure 1 has been submitted as a separate .tif file.

We understand that CONSORT discourages statistical comparisons of baseline characteristics, and ask you to remove this element from table 1.

This has been removed.

Please add a completed CONSORT checklist with your revision, labelled "S1_CONSORT_Checklist" or similar and referred to as such in your Methods section. In the checklist, please refer to individual items by section (e.g., "Methods") and paragraph number rather than by line or page numbers, as the latter generally change in the event of publication.

This has been added.

Comments from the reviewers:

*** Reviewer #1:

Statistical review

This paper reports a three arm randomised controlled trial testing SMS-based interventions for improving treatment of pregnant women with HIV. The trial was delivered successfully and was sizeable.

I had some comments on the statistical methods and reporting, listed below.

1.      Abstract: I realise that if the abstract is required to be 300 words (I was not aware PLOS medicine had this as a hard limit), then it may be difficult to address some of the following issues as more words would be required.

-       The authors mention an outcome being 'loss to follow up' - is this from the clinic or the trial?

We have clarified in the abstract that this is loss from clinical care, not the trial (page 2 line 10).

-       I would change 'no difference' to 'no significant difference' throughout. Since the trial was powered to testing the primary outcome it would be appropriate to add p-values in addition to the confidence intervals.

This has been added (page 2).

-       "Median ART adherence (100%) and incident ART resistance (1.48/100 py) did not differ by arm." - does this mean that incident ART resistance was exactly 1.48/100 py in each arm? Otherwise I would drop the figure and put 'significantly differ' instead of differ.

This has been corrected (page 2 line 16).

2.      Introduction, paragraph 2 - '1-way and 2-way arms' are used without much introduction (although it is briefly mentioned in the abstract).

We have added further explanation (page 5, final paragraph of introduction)

3.      Methods, randomisation and masking - this is well reported; can I just ask for the possible block sides to be added?

We have added this information to the Methods (page 7, randomization paragraph).

4.      Methods, outcomes - I'd recommend the pre-specified subgroups are added here.

This has been added (page 9, first paragraph of outcomes section).

5.      Methods, statistical analysis - when I first read this, I did not follow why the trial was powered on a composite criteria rather than the primary outcome itself. I think it is clearer once I read the protocol paper that this is separately the case for both outcomes. I would rewrite this a bit to ensure it is unambiguous.

This has been clarified (page 10, first paragraph of statistical analysis section).

6.      Methods, statistical analysis - overall there seemed to be some differences in the planned analyses between the protocol paper and this paper. The biggest difference is that the primary outcome appeared to be described as a time-to-event outcome in the protocol paper (with the sample size calculation framing it in terms of a hazard ratio). I would recommend the authors describe differences from the protocol and reasons.

Thank you for pointing out this discrepancy. After publication of the protocol paper, but before initiation of data analysis, we updated our analysis plan and the clinical trials registration record to use GEE rather than Cox regression. Our rationale was that GEE allowed us to use repeated measures per participant and maximize statistical power rather than only analyzing time to the first virologic non-suppression event. We have clarified this in the Methods (page 10, 1^st^ paragraph of statistical analysis section and viral non-suppression paragraph)

7.      Methods, statistical analysis - was any adjustment for multiple testing, due to the three arms, made?

We did not adjust for multiple testing due to the three arms.

8.      Methods, statistical analysis - what baseline characteristics were adjusted for?

We have added information that models were adjusted for primigravida and employment status (page 10, 1^st^ paragraph of statistical analysis)

9.      Methods, statistical analysis - I didn't follow here (or perhaps it could be made clearer in the outcomes) whether the analysis was the proportion of eligible visits where the participant was non-suppressed, or the proportion of participants who were non-suppressed at any point. If the former I would recommend ensuring it is clear whether the arm assignment influenced the number of visits per participant.

Thank you for pointing this out. It is the former; this has been clarified (page 10, viral non-suppression section of statistical analysis). We have also added a sentence to the results reporting that the number of observations per participant did not differ by arm (page 14, first paragraph of Outcomes).

10.     Methods, statistical analysis - I didn't follow what the actual data for retention and engagement in care was. Is it a count variable of number of scheduled clinic visits kept? Were number of scheduled visits consistent across arms?

Thank you for pointing this out. It is not a count, it is a binary repeated outcome defined as whether each scheduled clinic visits was attended on time (within 2 weeks of the scheduled date). We have clarified this in Methods (page 11, retention and engagement in care section). We have also added information about the number of scheduled visits per participant across arms in Results (page 14, 2^nd^ paragraph of outcomes section).

11.     Results, table 1 - generally it is not recommended to test for significant differences in baseline characteristics. Here the number of significant variables is as expected given the number of characteristics considered.

This has been removed.

12.     Results, outcomes of RCT - it's not too clear what the quoted adjusted risk ratios refer to (see comment 9).

We have added information that models were adjusted for primigravida and employment status (page 10, first paragraph of statistical analysis). This is also stated as a footnote in Table 2.

13.     Results/discussion - I note the significant 2-way vs 1-way result is highlighted in the discussion. I would ensure it is clear that there were a lot of significance tests reported and this (marginally) significant result could be a false positive due to multiple testing.

Thank you for pointing this out. We have added this to the discussion (page 21, first paragraph of discussion).

14.     Results, for the adherence data, when risk ratios are provided I'd clarify that this is referring to proportion of 'high adherence' periods.

This has been added to the adherence paragraph of the results (page 18).

15.     Abstract/Methods/results - were there any serious adverse events/harms reported in the study?

We have added a paragraph describing adverse events as the final paragraph of the results (page 20).

James Wason

*** Reviewer #2:

This is a very well written and conducted evaluation of SMS for a particularly important HIV risk group, pregnant and post-partum women.   The study is well reported and clearly well conducted and the findings are important. SMS is an increasingly popular method of communication with patient groups in Africa and is administered usually in a non-critical way. I think that this paper adds to the dialogue about recognizing both its utility and limitations.  I have only a few important points, that I list below:

1)      Perhaps in the introduction an again in the discussion there should be some recognition that in populations with high levels of the outcome of interest (say VL suppression), detecting treatment effects is difficult.

Thank you for this suggestion. We have added this to the discussion (page 21, 2^nd^ paragraph of discussion).

2)      This was non-blinded rather than unblinded.

This has been corrected (page 6, first paragraph of methods).

3)      Who actually administered the allocation to groups?  What I mean is, when a patient has agreed to be in the study, how does the enrolling staff member get from consent to allocation?

Study nurses administered allocation. This has been clarified in the methods (page 7, randomization and masking paragraph).

4)      I was a little surprised to see that the figures and tables were in the body of the manuscript. Did experienced writers see the final version?

The senior author reviewed the final version. We interpreted the journal guidelines to state that figures and tables needed to be inserted into the body of the manuscript. We have now submitted figures as separate .tif files.

5)      In the discussion, I think you should add a section on limitations of this RCT and also limitations of our knowledge of how to evaluate communication tools with patients. The SMS literature is, in general, not that clear in whether it is a useful tool or not and yet it is widely used among health programs across many settings in Africa. I happen to believe it is useful (but that is just a belief not necessarily driven by the evidence). Could you discuss some the challenges of evaluating digital technologies in such a rapidly changing digital world?  For example, you used SMS. But surely by the time this study was completed, people were using Whatsapp as their major form of communication? That's not a limitation, per se, but a challenge in such a rapidly moving environment.

We have highlighted limitations in the penultimate paragraph of the discussion (page 23). We have added a comment in the discussion on the lack of consensus about how to evaluate mHealth tools, understand mechanism and separate different components (page 21, 2^nd^ paragraph of discussion). We have added a comment to the discussion on use of SMS and its strength as an accessible platform despite the evolution of internet-based messaging (page 23, penultimate paragraph of discussion).

*** Reviewer #3:

Overall, this was a good paper that was generally clearly written, and with a thoughtful discussion. However, the study size was not large, and greater critique of the assumptions made for the study power calculation would be helpful.

Some specific comments, by section, are given below.

Abstract

1. Time on ART 1 year - does this mean follow-up from starting to 1 year after? Or on ART for 1 year at time of recruitment? It becomes clear in the main text, but needs to be clearer in the abstract.

This has been clarified (page 2).

2. Should report in the abstract the absolute values of the outcomes (percentages), and always by trial arm. For the purpose of the study, it is not so helpful to present figures pooled across trial arms and then say there was no difference by trial arm but not showing this.

We have added absolute outcome percentages by arm for the outcomes (page 2).

3. Related to the issue of study power, for which more detail should be provided in the methods of the main paper: the RR of 0.8 comparing 2-way SMS vs control for "ever virally non-suppressed" has a wide confidence interval, and it would be better to say "no evidence of a difference" rather than "no difference" compared to the control arm. I think this result does not correspond to the study power calculation, which was for the cumulative incidence of viral non-suppression by 24 months? It is important to present, as the first result, the one that corresponded to the primary study question for which the study was powered.

We have corrected the language used to describe lack of difference, to specify no significant difference. We have also clarified that the primary analysis was modified after publication of the protocol paper to be GEE, as presented in this paper. Cumulative incidence was used in the power calculation as a conservative approximation for power using GEE, acknowledging that repeated measures increase statistical power. In repeated measures sample size estimates we had power to detect smaller differences between trial arms than originally estimated. We now state this in the Methods (page 10, statistical analysis section).

4. Need to quote figures on the time to postpartum contraception, especially as the conclusion says the SMS made a difference -but not quantified in the results.

We have added information on contraception uptake and exclusive breastfeeding to 6 months postpartum to the results (page 20, 3^rd^ from last paragraph of results).

5. Not sure how the conclusion about targeted SMS follows from the results that are presented, the logic for this conclusion could be clarified in relation to the results presented.

Thank you for highlighting this. We have reorganized the 3^rd^ paragraph of the discussion to improve clarity (page 22).

Introduction

A bit more detail on the evidence to date on SMS to support adherence to ART post-partum would be helpful, beyond that there is "promising, but mixed evidence" and two papers cited. However, both the papers are systematic reviews - the introduction could mention that there are 2 systematic reviews, and then give a bit more information on what they found and whether there were patterns to the heterogeneity found e.g. did the SMS work better in particular settings, for particular groups of women, when implemented in particular ways?

Thank you for this suggestion. We have added more information in the first paragraph of the introduction (page 5).

Methods

1. Study power is very important here, given the study size is not large for a 3-arm trial and given the absence of evidence for effect on the primary outcomes. It seems from the methods text that the study was powered to be able to show a difference between 75% viral suppression in the control arm and 85% or more in the intervention arm, for a comparison of 2-year postpartum virologic non-suppression or retention. It could help to express this also as a risk ratio, given the analysis is done with risk (or rate) ratios, e.g. 15/25 = 0.6 so the study was powered to show a risk ratio of 0.6 (and then the risk ratios observed, were more of the order of 0.8 or 1/0.8 or closer to 1).

As previously noted, we have added details about our rationale for changing analysis approach, including new sample sizes estimates that demonstrate power to detect smaller associations. We have added more details to the statistical power section of the Methods (page 10).

Please clarify if this outcome was "virologic non-suppression at the 2 year time point" or "any virologic suppression during 2 years of follow-up". This matters, for which results correspond directly to the study power calculation. Please clarify exactly which analysis in the results corresponds to this power calculation (make sure the language in the results aligns precisely with the language for the study power calculation).

This has been clarified in the statistical power and viral non-suppression sections of the methods (page 10).

2. Page 14 - says qualitative evaluation of participant experiences will be reported elsewhere, but something would be useful in this paper to explain lack of evidence of effect of the SMS. Please consider including some reference to what was learnt from the qualitative evaluation, in the discussion even if not in the results.

Analysis of the post-RCT qualitative findings is ongoing so we are unfortunately not yet able to share findings.

3. Page 15 - Please explain a bit more on "Anderson-Gill regression". Cumulative incidence could have been estimated using Kaplan-Meier methods and using Cox regression to compare trial arms; please explain how "Anderson-Gill regression" is different for a "time to event" analysis and why it was used instead of Kaplan-Meier estimates of the percentage with the outcome by different time points and Cox regression to estimate rate ratios. There is a footnote/caption to Figure 2 which explains why Anderson-Gill regression was used, and that it gave similar hazard ratios to Cox regression of "time to first event", but this information should be in the Methods.

Similar to the rationale for GEE, Andersen-Gill regression enables comparison of repeated viral non-suppression events rather than time to first non-suppression. This enables more statistical power to detect differences in episodic non-suppression events rather than a single time to event. In response to other reviewer comments above and because the previously published protocol paper specified Cox regression as the analytical approach, we have modified Figure 2 and Table 2 to present Cox regression and include Andersen-Gill regression results in Supplementary Table 1. Note that we also updated Figure 2 to restrict the x-axis to the period of follow-up included in analysis (the initial submission included a previous version of the figure in error).

Results

1. Please present first the results that correspond directly to the study power calculation. It is reported in the first paragraph of the "outcomes of RCT" paragraph that overall 20% were ever non-suppressed by 2 years - if this is the primary outcome, please report it in the text separately by trial arm. But, in Table 2 the focus is on (1) viral non-suppression at any time point (regardless of time point) and (2) viral suppression by particular time points i.e. 6, 12, 24 months post-partum.
Please consider some re-ordering of the presentation of results, to first report on the primary outcomes defined in the study power calculation, and then to present other results that are informative.

Thank you for pointing out this inconsistency between our Methods and Results. We have clarified in the Methods that the primary analysis was having non-suppression at any time during follow-up (page 10, viral non-suppression paragraph) and rearranged the results so that this comparison is presented first (page 14, first paragraph of outcomes of RCT).

2. Please clarify how the numerators and denominators are counted for outcome (2) [viral suppression by particular time points] in Table 2, as the model used is a log-binomial model; is the denominator for the 24-month time point all individuals who were followed up at 24 months?

The denominator is the cumulative number of people who have any VL result available after enrollment, within the follow-up period. We have added a footnote to Table 2 and additional details to methods regarding the denominator for the cumulative incidence analysis (page 10, viral non-suppression section).

3. Confidence intervals are in general wide, e.g. aRR 0.80 [0.52-1.23], p=0.31 for comparing 2-way SMS with control for non-suppression over time (page 20). The wide confidence intervals, and how they relate to study power, could be given more attention in the discussion.

Thank you for this helpful comment. We agree and have added a comment about this in the penultimate paragraph of the discussion (page 24).

4. Page 20 - Outcomes of RCT. Please report in the text the percentage with cumulative incidence of virologic suppression by 24 months, as well as the risk ratios. There are numerators and denominators for the log-binomial model used to estimate cumulative incidence of viral non-suppression in Table 2, but it is not completely clear how these numbers are counted - who contributes to the denominator, and who contributes to the numerator? - it is important that is clear in both Table 2 (e.g. with footnotes for more detail) and in the text.

We have added the percentages to the results (page 14, first paragraph of outcomes section). We have added a footnote to Table 2 and additional details to methods regarding the denominator for the cumulative incidence analysis (page 10, viral non-suppression section).

5. Was it considered to use the Kaplan-Meier method, rather than a log-binomial model, for a simple and transparent unadjusted analysis of the comparison of cumulative incidence of viral non-suppression by trial arm that allows for loss to follow-up? And then to use Cox regression to estimate hazard ratios to compare trial arms? It would help to understand the choice of the log-binomial model, if the numerators and denominators were precisely defined for each time point (who contributes to the analysis), see comment also above.

Cox regression has now been added to Table 2.

Figure 2A shows "time to first non-suppression", I think from a Kaplan-Meier analysis, but then presents hazard ratios from Anderson-Gill regression that allow for repeated instances of viral non-suppression. I think it would be better for Figure 2A to show the hazard ratios from Cox regression, that directly correspond to the graph. The findings from the Anderson-Gill regression could be included in a table instead - they should be included as part of Table 2, and I think they correspond closely to the primary analysis defined for the study power calculation (cumulative incidence of viral suppression by 24 months) but are not exactly the same as they allow for repeated events (of viral non-suppression).

We have replaced the Andersen-Gill findings with Cox regression findings in Figure 2A and in the Results (outcomes of RCT section). We have also added these findings to Table 2. Andersen-Gill findings are presented in Supplementary Table 1.

6. In Table 2, please write out what RR stands for in the table column heading, as it matters for interpretation whether is a risk ratio (which I think it is) or a rate ratio and it needs to be clear for the reader and correspond to the analysis that was done. I think from the log-binomial models, it is a risk ratio, but for example if Cox regression were done it would be a rate ratio / hazard ratio.

A footnote in Table 2 specifies that RR refers to relative risk.

Thank you for your consideration for our manuscript for publication in PLOS Medicine. We look forward to hearing from you.

Sincerely,

Keshet Ronen

Acting Assistant Professor

Department of Global Health

University of Washington
