# Supplementary material for: SMS messaging to improve retention and viral suppression in prevention of mother-to-child HIV transmission (PMTCT) programs in Kenya: A 3-arm randomized clinical trial
Source: PLoS Med. 2021 May 24;18(5):e1003650. doi: 10.1371/journal.pmed.1003650 (PMC8186790; doi:10.1371/journal.pmed.1003650)
Supplement: Attachment — Submitted filename: ResponseToReviewers2_final.docx [file pmed.1003650.s006.docx]

May 6, 2021

Dear Dr. Turner,

Re: PMEDICINE-D-21-00822R1

Thank you for your careful review of our manuscript. We are delighted that you expect to be able to accept our paper. We have addressed the reviewers’ and editors’ comments below.

Requests from Editors:

At line 19 (abstract), please make that "infant HIV infection".

This has been corrected.

At line 22, please adapt the text to "... but was associated with improved initiation of postpartum contraception" or similar, as this result is apparently from an exploratory analysis.

This has been corrected.

At line 23, we suggest "women are virally suppressed".

This has been corrected.

At line 27 (author summary), please make that "previously reported apparent effect" or similar, as noted above.

This has been corrected.

Please make randomized/randomised and derivatives consistent through the paper.

We have standardized the paper to use US spelling.

Please remove the competing interest statement from the end of the main text.

This has been removed.

Please make that "PLoS ONE" in reference 29.

This has been corrected.

Please adapt the attached CONSORT checklist so that individual items are referred to by section (e.g., "Methods") and paragraph number. Page and line numbers should not be used, as these generally change in the published paper.

This has been corrected.

Comments from Reviewers:

*** Reviewer #1:

Thank you to the authors for addressing all my previous comments well. I have no further issues to raise.

We are grateful for the reviewer’s feedback.

*** Reviewer #2:

The authors have done a very good job addressing all of the reviewer's comments and suggestions. They should be commended for the hard work necessary to conduct and successfully complete this trial.

We are grateful for the reviewer’s feedback.

Thank you for your consideration for our manuscript for publication in PLOS Medicine. We look forward to hearing from you.

Sincerely,

Keshet Ronen

Acting Assistant Professor

Department of Global Health

University of Washington
